# Small molecule inhibitors of cyclin-dependent kinase 9 for cancer therapy

**DOI:** 10.1080/14756366.2021.1890726

**Published:** 2021-02-25

**Authors:** Aisha Alsfouk

**Affiliations:** Department of Pharmaceutical Sciences, College of Pharmacy, Princess Nourah bint Abdulrahman University, Riyadh, Saudi Arabia

**Keywords:** CDK9, proliferative disorders, kinases

## Abstract

Cyclin-dependent kinase 9 (CDK9) plays a vital role in transcription through regulation of short-lived anti-apoptotic genes required for cancer cell survival. Therefore, targeting CDK9 with small molecule inhibitors has emerged as a potential cancer therapy. This article reviews the most recent CDK9 patent literature (2012–2020) related to small molecule inhibitors in cancer along with their selectivity profile and biological results in preclinical studies.

## Introduction

Since the discovery of imatinib in 2001, the first kinase inhibitor to obtain Food and Drug Administration (FDA) approval for treatment of chronic myeloid leukaemia, there has been a great interest in kinases as therapeutic target in cancer, especially for those malignant conditions that currently have limited treatment options.

Cyclin-dependent kinases (CDKs) form heterodimers with a specific family of proteins called cyclins. These functional CDK–cyclin complexes regulate cell cycle progression and gene transcription^1^. CDK9 is a member of the CDK family; it dimerises with cyclin T to form the positive transcription elongation factor b (p-TEFb) complex^2^^,^^3^. This complex stimulates transcription elongation through phosphorylation of the C-terminus domain (CTD) subunit of RNA polymerase II at Ser2^4^. CDK9 plays a vital role in controlling the transcription of a number of genes, including Myc, a proto-oncogene that regulates processes required for cell growth and cell cycle progression, and Mcl-1, an anti-apoptotic member of the Bcl-2 family that enhances cell survival^5^. Therefore, CDK9 inhibition reduces messenger RNA (mRNA) transcription and prevents the expression of target genes (e.g. Myc and Mcl-1), which together regulate proliferation and cancer cells survival.

There is a sufficient evidence to support CDK9 as a valid therapeutic target in cancer through promotion of cell proliferation and regulation of anti-apoptotic proteins such as Mcl-1 and Myc that initiate cancer cell immortality. A dysregulated CDK9-related pathway has been established as a major component for initiation and/or progression of a number of malignancies, including lymphomas, prostate cancer, breast cancer and others^5–11^. Multiple studies have reported that dysregulated CDK9 signalling is associated with pathogenesis of a number of haematological malignancies^9^^,^^11^^,^^12^. Elevated Mcl-1 expression has been linked to the development of acute myeloid leukaemia in human cells^13^. High p-TEFb activity has been found in a number of pathological diseases, such as lymphomas and Hodgkin’s disease^14^. CDK9 plays a vital role in prostate cancer. Initially, androgens stimulate growth and survival of prostate cells, and most castration-sensitive prostate cancer responds to androgen deprivation. However, 20% of prostate cancer patients develop castrate-resistant prostate cancer (CRPC), which is unresponsive to conventional therapy and associated with a poor prognosis. Recently, CDK9 has been identified as a key component in CRPC through modulating the activity of the androgen receptor^6^^,^^7^^,^^9^^,^^15^. Although there is a diverse genetic background in breast cancer, alteration in CDK9 expression is one molecular pathway in the development of the disease through its interaction with proto-oncogenes^16^. A study found that miR-874 plays an important role in breast cancer by inhibiting proliferation and inducing apoptosis and cell cycle arrest. In this study, CDK9 was a direct target of miR-874, which negatively regulates its proteins levels^17^. A study found that the proto-oncogene Myb expression in oestrogen receptor–positive breast cancer is downregulated by CDK9. In this study, the role of CDK9 was reinforced by using CDK9 inhibitors, e.g. SNS-032, CDKi and CAN-508. These data demonstrated an increase in tumour cell apoptosis and preventing cell growth^18^.

## CDK9 clinical applications

**Figure 1.**
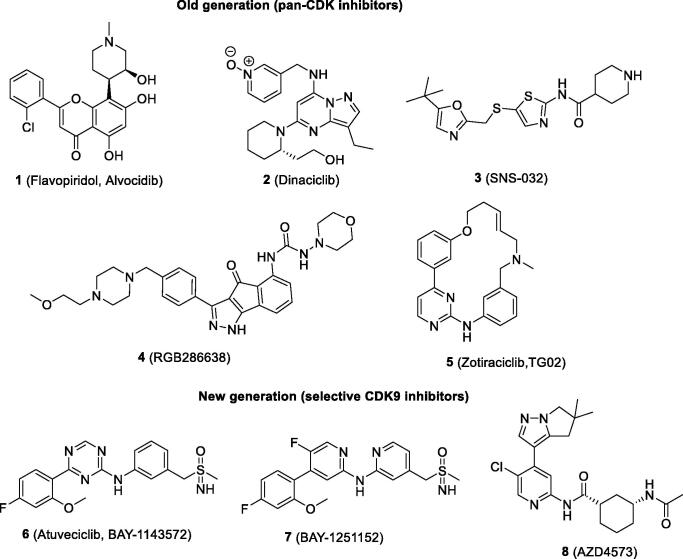
CDK9 inhibitors that have been or are being evaluated in clinical trials.

Since CDK9 was identified as a promising therapeutic opportunity in cancer, its inhibition has become a main strategy for large pharmaceutical companies, and a number of chemical motifs have been developed. These inhibitors commonly function as ATP competitive inhibitors and have low molecular weight and drug-like properties. Several of these molecules have progressed into clinical trials as anti-proliferative agents for the treatment of various types of cancer^12^^,^^19–27^. The first generation of inhibitors evaluated in clinical trials were pan-CDK inhibitors, which inhibit CDK9 as well as other CDK isoforms and other kinases (Figure 1 and Table 1). Flavopiridol **1** was the first CDK inhibitor to enter clinical trials. Although it inhibits other CDK isoforms, including CDK4, CDK5 and CDK7, its primary anti-tumour mechanism is now understood to be through transcriptional regulation via CDK9/P-TEFb^37^. Its evaluation in phase II clinical trials for treatment of leukaemia showed up to 58% complete response, but with a high incidence of adverse effects, with up to 87% high risk^19^^,^^24^^,^^38^^,^^39^. Dinaciclib **2** is a potent pyrazolopyridine inhibitor of CDK9, CDK2, CDK5, CDK7 and CDK1 with low nanomolar IC_50_ values. In *in vitro* studies, it inhibits Rb phosphorylation and blocks incorporation of thymidine DNA^40^. In three phase II clinical trials for the treatment of breast cancer, lung cancer and acute leukaemia, there was no complete response to treatment and there was a high incidence rate of adverse effects in up to 95% of the patients^41–43^. SNS-032 **3** is a thiazole carboxiamide derivative with a potent CDK9 inhibitory effect (IC_50_=4 nM) and some activity against CDK2 and CDK7. In acute myeloid leukaemia cell lines, **3** inhibits RNA polymerase II phosphorylation, supresses Mcl-1 and XIAP and induces apoptosis^44^. It has been evaluated in phase I studies in subjects with leukaemia, but its clinical results were limited due to grade 3 and 4 toxicities, mainly myelosuppression^32^^,^^45^. RGB286638 **4** is a pan-CDK inhibitors with a low nanomolar IC_50_ against several CDKs. Its evaluation in human trials demonstrated the lack of a complete response to the treatment, and 23% of the patients exhibited adverse effects^22^. Zotiraciclib (TG02 **5**) is a macrocyclic compound bearing phenyl-2-aminopyrimidine. It a multi-target CDK inhibitor – including CDK9. In a phase I study, when combined with temozolomide for treatment of recurrent malignant gliomas, it showed a tolerable toxicity profile; investigations have progressed into a phase II trial (NCT02942264)^46^. Notably, the majority of the aforementioned inhibitors (**1**–**5**) showed limited success with regard to treatment with high rates of adverse effects. These outcomes may be due to the lack of selectivity for CDK9.

**Table 1.** Selectivity profile of CDK9 inhibitors that have been or are being evaluated in clinical trials (IC_50_ are presented in nM).

Inhibitor	CDK9	CDK2	CDK5	CDK7	CDK4	References
Old generation (pan-CDK inhibitors)						
Flavopiridol (**1**)	11	282	110	514	132	^28–30^
Dinaciclib (**2**)	4	1	1	–	–	^28^ ^,^ ^31^
SNS-032 (**3**)	4	48	340	62	925	^28^ ^,^ ^32^
RGB-286638 (**4**)	1	3	5	44	4	^30^ ^,^ ^33^
Zotiraciclib (**5**)	3	5	4	37	–	^34^ ^,^ ^35^
New generation (selective CDK9 inhibitors)						
Atuveciclib (**6**)	6	1000	1600	>10000	–	^26^
BAY-1251152 (**7**)	4	2920	–*	–*	–*	^36^
AZD4573 (**8**)	3	–ᶧ	–ᶧ	–ᶧ	–ᶧ	^27^

CDK: cyclin-dependent kinase; IC_50_: half maximal inhibitory concentration. *no specific IC_50_ value was disclosed, but it is claimed to be 50 fold greater than the CDK9 IC_50_. ᶧno specific IC_50_ value was disclosed but it claimed to be 10 fold greater than CDK9 IC_50_.

The second generation of inhibitors evaluated in clinical trials are selective CDK9 inhibitors. In 2017, researchers at Bayer identified the aminotriazine derivative atuveciclib (BAY-1143572, **6**) as the first highly selective CDK9 inhibitor. In a biochemical assay, it is highly potent against CDK9 (IC_50_=6 nM) and selective over other CDK isoforms (>150 fold)^26^. In an adult T-cell leukaemia/lymphoma model, it inhibits RNA polymerase II phosphorylation and induces apoptosis through Myc and Mcl-1 depletion^47^. It is currently being evaluated in phase I clinical trials for treatment of advance acute leukaemia (NCT02345382)^48^. The same research group has also identified a second selective and potent CDK9 inhibitor, BAY-1251152 **7**, an aminopyridine derivative structurally related to **6**. This compound presents similar biological results (CDK9 IC_50_=4 nM and selectivity >50 fold over other CDKs). It is currently being evaluated in a phase I clinical trial for treatment of acute leukaemia (NCT02635672)^36^^,^^49^. A novel and potent aminopyridine derivative, AZD4573 **8**, has been identified by researchers at AstraZeneca. It shows an CDK9 IC_50_ value of 3 nM and selectivity over other CDKs and kinases claimed to be >10 fold. In a haematological tumour model, it induces apoptosis through suppression of Mcl-1 expression^50^. It is currently being evaluated in a phase I clinical trial in patients with refractory haematological malignancies (NCT03263637)^51^.

## The new CDK9 patent literature

Patents were collected from world intellectual property organisation (WIPO), Espacenet and Scifinder databases using “CDK9” and “cancer” as keywords and combining the results. Patents that did not cover small molecule inhibitors or not related to cancer as well as duplicated documents were manually excluded. The selected patent applications were classified according to structural similarity of their subject compounds in to seven structure classes. Patent documents in languages other than English were translated to understand the inventions’ claims.

### 2-Aminopyridines/pyrimidines

In 2018, GenFleet Therapeutics filed a patent on 5-chloro-2-amino-pyridines as potent and selective CDK9 inhibitors (patent in Chinese) for treatment of haematological malignancies (Table 2). Compounds **9** and **10** exhibit CDK9 inhibition IC_50_ values of 0.93 and 1.27 nM, respectively. In addition, they show selectivity at least 1000 fold over other CDK isoforms and high selectivity over a panel of 468 kinases^52^. In cell-based assays, **9** and **10** show good selectivity for cancer cells: they exhibit strong cytotoxicity against leukaemia and lymphoma cell lines (average IC_50_ values of 31 nM) with no effect on normal cell lines (CHL and CHO). In acute myeloid leukaemia (AML) cell lines (HL-60, OCI-AML-3 and MV4-11) and the acute promyeloid leukaemia cell line NB-4, compound **9** presents concentration-dependent inhibition of CDK9 phosphorylation through downregulation of RNA polymerase II, Mcl-1 and c-Myc. In addition, it induces caspase-3 protein cleavage by poly (ADP-ribose) polymerase (PARP) and blocks the cells in G0–G1 phase in the above-mentioned four cell lines. In the MV4-11 mouse model, **9** reduces the tumour weight by 98.7% with a high dose (20–30 mg/kg), but this high dose shows a negative effect on Balb/c mouse body weight. However, with the low dose of 10 mg/kg, the tumour weight is significantly reduced, the body weight of the mice remains stable during the treatment and the compound is well tolerated^53^.

**Table 2.** 5-Chloro-2-aminopyridines CDK9 inhibitors published by GenFleet therapeutics.

	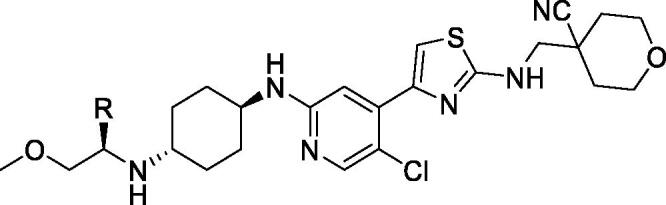	
Compound	9	10
*R*	H	Me
IC_50_ (nM)		
CDK9/cyclin T1	0.93	1.27
CDK2/cyclin A	6850	2860
CDK5/p25	6950	4640
CDK7/cyclin H	3700	1720
Cytotoxicity*	31	30

CDK: cyclin-dependent kinase; IC_50_: half maximal inhibitory concentration. *Average of 11 cell lines.

In 2013, researchers at Changzhou Le Sun Pharmaceuticals disclosed 2-aminopyrimidines (**11**–**13**) for treatment of proliferative disorders (Table 3). Compound **11** (CDKI-73) inhibits CDK9 as well as other CDK isoforms, including CDK1, CDK2 and CDK5. It also shows a low nanomolar inhibitory effect towards Aroura A, Aroura B and GSK3β. In MTT proliferative assay, it shows a cytotoxicity IC_50_ of 30 nM in MCF-7 and HCT-116 cell lines. In primary CLL cells, CDKI-73 shows a LD_50_ of 80 nM using an apoptosis assay with no effect on normal B cells and T cells. In pharmacokinetic measurements in mice, it exhibits 56% oral bioavailability following a 10 mg/kg oral dose^54^. In combination with fludarabine, it shows strong synergistic effect on 48 h cytotoxicity assay using CLL cells. Also, the combination markedly represses Bcl-2, Mcl-1, XIAP, CCND1 and CCNDD2 gene expression. Furthermore, the synergistic combination retains the pro-survival, CDK40L-expression co-culture condition, which is known condition to induce resistance to fludarabine^55^.

**Table 3.** 2-Aminopyrimidines CDK9 inhibitors published by Changzhou Le Sun Pharmaceuticals.

	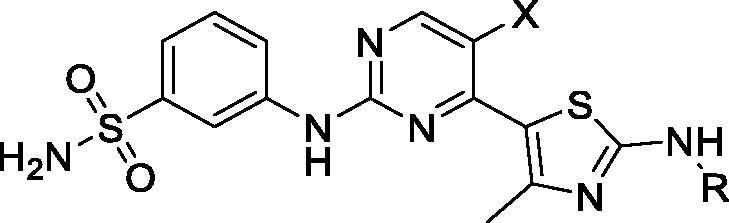		
Compound	11 (CDKI-73)	12	13
R	Me	H	Me
X	F	F	Cl
*K*_i_ (nM)			
CDK9/T1	4	3	10.5
CDK1/B	4	7	19
CDK2/A	3	3	10
CDK5/p35	0.5	1.5	1.5
CDK6/D3	167	116	87.5
CDK7/H	91	87	695
GI_50_ (nM)			
Cytotoxicity*	30	225	75

CDK: cyclin-dependent kinase; GI_50_: half maximal inhibition of cell proliferation; K_i_: inhibitory constant. *Average of two cell lines.

**Figure 2.**
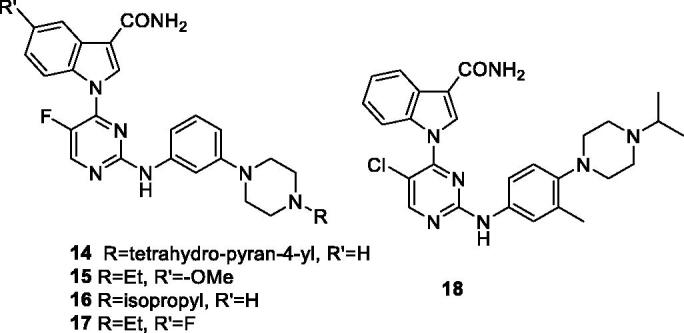
2-Aminopyrimidines CDK9 inhibitors published by Ancureall Pharmaceuticals.

In 2019, Si et al. at Ancureall Pharmaceuticals filed a patent on 2-aminopyrimidines (patent in Chinese). A total of 172 compounds were prepared and assayed against CDK9 (the most interesting examples **14**–**18** are shown in Figure 2). No specific IC_50_ values were disclosed but it claimed to be <10 nM against CDK9 in biochemical assay and <1 nM against cell growth in cell-based assay using 44 CDK9-expressing tumour cell lines, including human acute leukaemia MOM13 cells. In *in vivo* animal studies, compound **14** effectively inhibits tumour growth by 91.3% at dose of 50 mg/kg with no effect on the body weight of the mice^56^.

**Figure 3.**
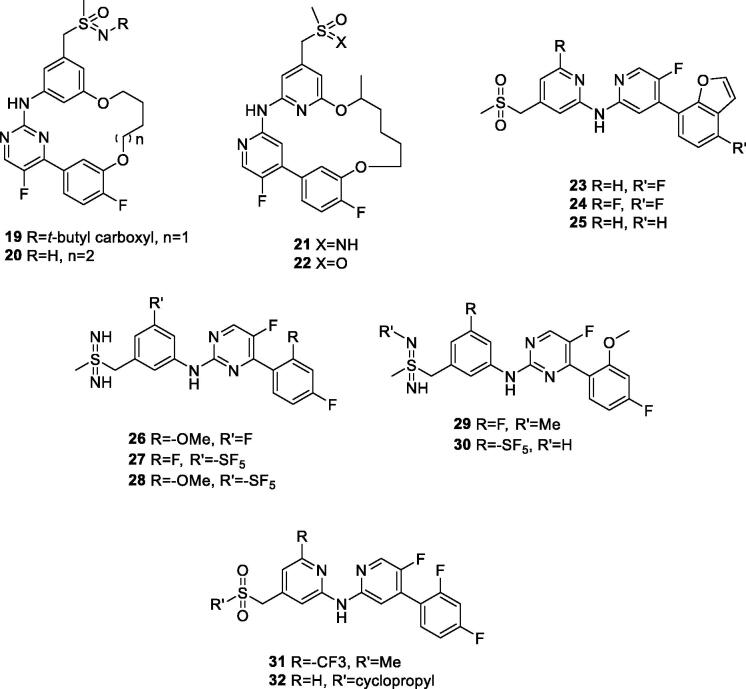
2-Aminopyridines/pyrimidines CDK9 inhibitors published by Bayer.

Bayer filed 10 patent applications during 2013–2018 covering 2-aminopyridines/pyrimidines as selective CDK9 inhibitors for cancer therapy. In three publications, Lucking et al. presented disubstituted 5-fluoropyrimidine compounds bearing a sulfondiimine and sulphone group (representative examples are shown in Figure 3 and Table 4). These compounds show CDK9 IC_50_ values between 4 and 51 nM and CDK2 IC_50_ between 190 and 2300 nM. In cytotoxicity assay, these examples exhibit IC_50_ between 35 and 1270 nM in eight cell lines. In pharmacokinetic assays, these derivatives exhibit thermodynamic aqueous solubility between 1.2 and 259 mg/L at pH 6.5. In a Caco-2 permeability test, these examples show an efflux ratio between 1.1 and 6.9^60^^,^^62^^,^^63^. In follow-up publications, the same group claimed macrocyclic 2-aminopyridine/pyrimidines with improved potency and selectivity towards CDK9. A total of 32 derivatives were prepared and assayed against CDK9 and CDK2. The most potent and selective examples are shown in Table 4. These examples have reported single digit nanomolar CDK9 IC_50_ values in *in vitro* assay and selectivity exceeds 1000 fold over CDK2. These examples exhibit low nanomolar IC_50_ values in proliferation assay using eight cancer cell lines^57^^,^^58^. In separate publications, the same group disclosed five 5-flouoro-N-(pyridyl-2-yl) pyridine-2-amine derivatives (Table 4) as potent CDK9 inhibitors and selectivity no greater than 29 fold over CDK2. In cytotoxicity assay in seven cell lines, these examples show low nanomolar IC_50_ values^59^^,^^61^^,^^64–66^. An agent from these patents has progressed into clinical trials (BAY-1251152, **7**) for treatment of leukaemia, as described in the “CDK9 clinical applications” section (see Table 1).

**Table 4.** 2-Aminopyridines/pyrimidines CDK9 inhibitors published by Bayer.

Compound*	IC_50_ (nM)			mg/L	Efflux ratio in Caco2 permeability assay	Ref
	CDK9	CDK2	Cytotoxicityᶧ	Aqueous solubility		
**19**	5	>20000	160	ND	ND	^57^
**20**	5	16200	157	ND	ND	^57^
**21**	2	2450	NT	ND	ND	^58^
**22**	3	3590	55	ND	ND	^58^
**23**	3	87	25	ND	ND	^59^
**24**	4	71	33	ND	ND	^59^
**25**	4	69	77	ND	ND	^59^
**26**	13	790	370	155	5.6	^60^
**27**	17	2300	309	1.2	1.1	^60^
**28**	4	400	142	2.2	1.6	^60^
**29**	15	1300	682	97	ND	^60^
**30**	4	400	140	2.2	1.6	^60^
**31**	20	20000	1050	ND	ND	^61^
**32**	13	410	3000	ND	ND	^61^

CDK: cyclin-dependent kinase; IC_50_: half maximal inhibitory concentration; ND: not determined. *From corresponding structures in Figure 3. ᶧAverage of eight cell lines.

Wang et al. at Aucentra Therapeutics filed a patent on 2-aminopyrimidines bearing an imidazopyridine group. A total of 181 compounds were prepared and assessed against CDK9 and other CDK isoforms. Compounds **33** and **34** are representative examples from this series; they inhibit CDK9 with *K*_i_ values of 6 and 8 nM, respectively, and selectivity greater than 40 fold over other CDKs (Table 5). In a cell viability assay, they exhibit anti-proliferative activity with GI_50_ in the sub-micromolar range in 12 cancer cell lines including prostate cancer, breast cancer, ovarian cancer and leukaemia cell lines^67^.

**Table 5.** 2-Aminopyrimidines CDK9 inhibitors published by Aucentra Therapeutics.

	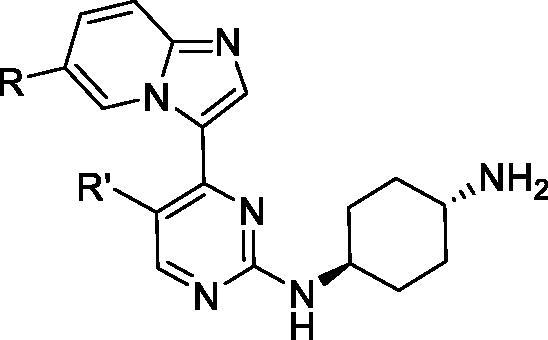	
Compound	33	34
*R*	3-Fluoroailin-1yl	Ph
*R*′	F	Me
*K*_i_ (nM)		
CDK9/T1	6	8
CDK1/B	70420	101530
CDK2/A	240	430
CDK4/D1	691	2620
CDK6/D3	1710	1540
CDK7/H	730	600
GI_50_ (nM)		
Cytotoxicity*	0.24	0.61

CDK: cyclin-dependent kinase; GI_50_: half maximal inhibition of cell proliferation; K_i_: inhibitory constant. *Average of 12 cell lines.

**Figure 4.**
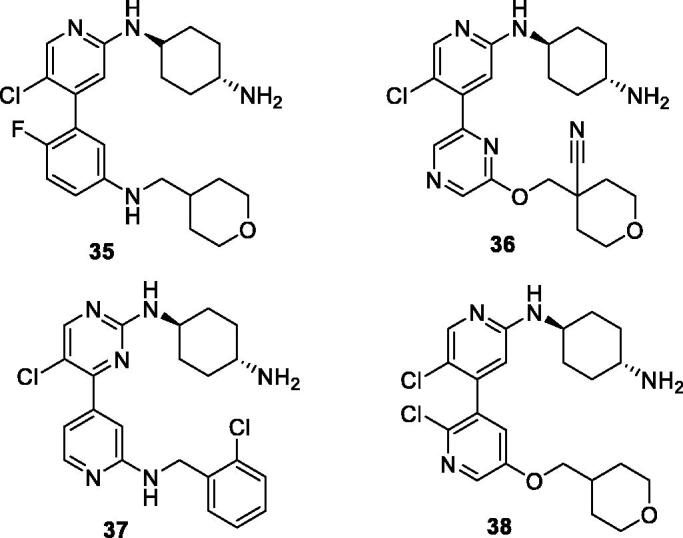
4-Aryl/hetroaryl-2-aminopyridines/pyrimidines CDK9 inhibitors published by Novartis.

Throughout 2012, Novartis filed four patent applications claiming 2-aminopyridines/pyrimidines bearing a 4-aryl/heteroaryl group as CDK9 inhibitors. The first patent application described 4-phenyl-2-aminopyridines. A total of 108 compounds were designed and assessed against CDK9 (see representative example **35**; Figure 4). These inhibitors generally display a low nanomolar IC_50_ value towards CDK9^68^. A second patent disclosed a large set of biaryl systems (111 compounds in total including **36**; Figure 4). These compounds show very strong activity against CDK9; the IC_50_ can reach 0.5 nM^69^. The third patent disclosed 4-pyridin-4-yl-2-aminopyrimidines (39 compounds including **37**; Figure 4), many of which are highly potent^70^. The final patent describes 2-aminopyridines with 4-pyridin-3yl group (total of 92 compounds e.g. **38**; Figure 4)^71^.

### Other heteroaryl compounds

**Figure 5.**
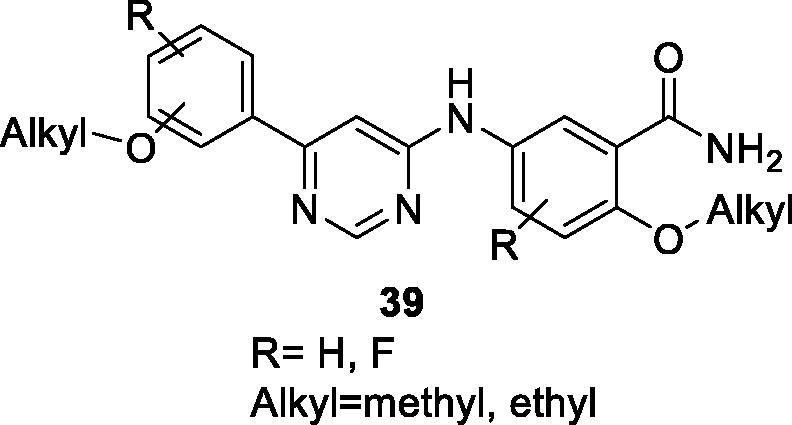
4-Aminopyrimidines CDK9 inhibitors published by ViroStatics.

In 2014, researchers from ViroStatics have filed a patent on 4-aminopyrimidines as CDK9 inhibitors and assessed their antiviral and anti-proliferative effects (patent in Japanese). The patent includes 148 compounds with the general formula **39** (Figure 5), most of which show CDK9 IC_50_ value <10 µM and supress the viability and proliferation of tumour cells with IC_50_ of 0.4 µM^72^.

AbbVie has been the main contributor to CDK9 patent literature in the last decade; they have mainly focussed on anti-proliferative activity. A wide array of scaffolds have been studied, including pyrrolopyridines/pyrimidines, tetracyclic systems and pyridines. In 2014, Lai et al. published a patent application on pyridines as CDK9 inhibitors. A total of 339 compounds were tested in a CDK9 assay and cancer cells. Compounds **40** and **41** are representative examples from this series (Table 6); the reported CDK9 IC_50_ are 27 and 150 nM, respectively. Their cytotoxicity IC_50_ are 0.22 and 0.96 µM, respectively (average of two cell lines). Both compounds were assayed *in vivo* using H929 human multiple myeloma cancer xenograft model. They show up to 48% tumour growth inhibition with a low dose of 3.75 mg/kg and up to 70% tumour growth inhibition with a high dose of 15 mg/kg^73^.

**Table 6.** Pyridines CDK9 inhibitors published by AbbVie.

	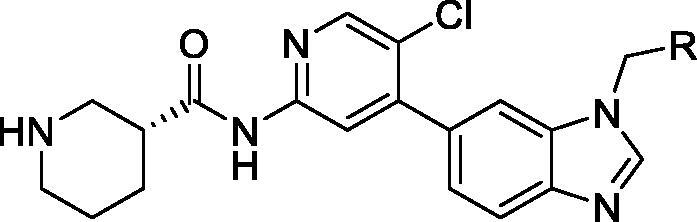	
Compound	40	41
*R*	3-Fluorophenyl	Morpholin-4yl
CDK9 IC_50_ (nM)	27	150
Cytotoxicity* IC_50_ (µM)	0.22	0.96

CDK: cyclin-dependent kinase; IC_50_: half maximal inhibitory concentration. *Average of two cell lines.

**Figure 6.**
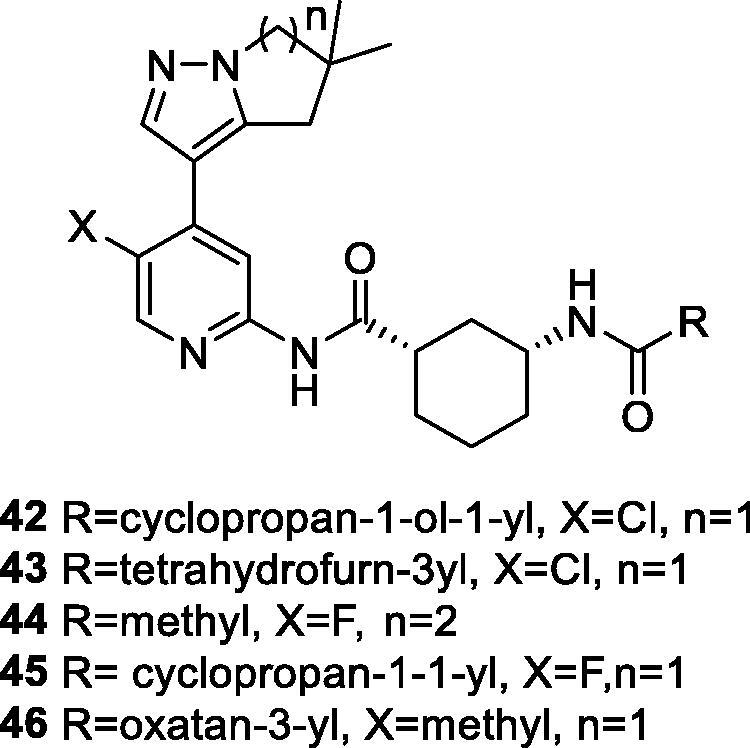
Pyridines CDK9 inhibitors published by AstraZeneca.

In 2017, researchers at AstraZeneca filed a patent on pyridine and pyrimidine amide derivatives as inhibitors of CDK9, with potential uses in hyper-proliferative diseases. Around 83 compounds were prepared and assayed in a CDK9 assay with two ATP concentrations (at Km and a high concentration of 5 mM). Several of the compounds show a single digit nanomolar CDK9 IC_50_ at both ATP concentrations. The phosphorylation of RNA polymerase II at Ser2 by the inhibitors were determined in the breast cancer cell line MCF7. The majority of the compounds show a sub-micromolar IC_50_ against phosphorylated RNA polymerase II Ser2. The induction of caspase activity was measured after 6-h treatment of myeloid leukaemia MV411 cells with the compounds; the IC_50_ reaches 10 nM in this assay. The most potent examples are shown^74^ in Figure 6 and Table 7. An agent from this series is currently being evaluated clinical trials (AZD4573, **8**) for treatment of haematological malignancies, as described in the “CDK9 clinical applications” section (see Table 1).

**Table 7.** Pyridines CDK9 inhibitors published by AstraZeneca.

Compounds*	CDK9 (IC_50_, nM) ATP conc. At Km	CDK9 (IC_50_, nM) high ATP conc.	Phospho-RNAPolyII Ser2 (IC_50_, nM)	Caspase activity MV4-11 (IC_50_, nM)
**42**	<3	<3	<7	13
**43**	<3	<4	11	15
**44**	<3	<3	10	15
**45**	<3	<3	7	10
**46**	<3	9	38	36

CDK: cyclin-dependent kinase; IC_50_: half maximal inhibitory concentration; Phospho-RNAPolyII: phosphorylated RNA polymerase II. *From the corresponding structures in Figure 6.

**Figure 7.**
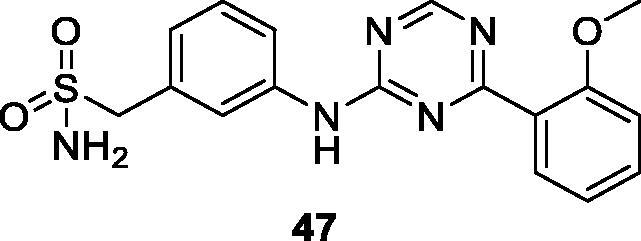
A 2-aminotriazine CDK9 inhibitor published by the Lead Discovery Centre.

The Lead Discovery Centre claimed the 2-aminotriazine derivative **47** as a potent and selective CDK9 inhibitor for treatment of cancer (Figure 7). Compound **47** exhibits CDK9 inhibitory activity with an IC_50_ of 1 µM and selectivity greater than 30 fold against other CDKs, including CDK2, CDK1, CDK4, CDK6 and CDK7, and greater than 50 fold across a kinase panel. In cell-based assay, it shows anti-proliferative activity with IC_50_ in sub-micromolar range against various cell lines^75^.

Bayer filed a patent on 2-aminotriazines as CDK9 inhibitors. A total of 91 compounds were prepared and assessed against CDK9 and CDK2 (representative examples are shown in Table 8). These compounds show CDK9 IC_50_ values between 2 and 30 nM and selectivity greater that 100 fold over CDK2. In cytotoxicity assays, these examples exhibit IC_50_ in sub-micromolar range in six cell lines. In pharmacokinetic assays, these derivatives exhibit good thermodynamic aqueous solubility up to 1200 mg/L at pH 6.5^76^. An agent from this patent has progressed into clinical trials (atuveciclib, BAY-1143572, **8**) for treatment of leukaemia, as described in the “CDK9 clinical applications” section (see Table 1).

**Table 8.** 2-aminotriazines CDK9 inhibitors published by Bayer.

	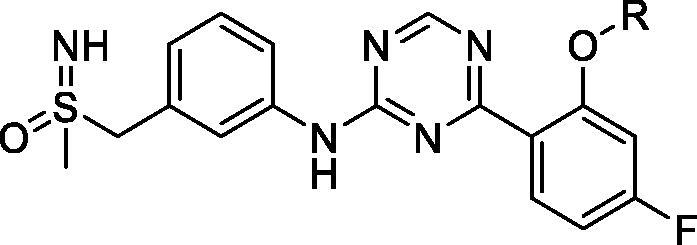	
Compound	48	49
*R*	1,2-oxazol-3yl	3,4-difluorobenzen-1yl
IC_50_ (nM)		
CDK9	7	11
CDK2	2000	1300
Cytotoxicity*	680	715

CDK: cyclin-dependent kinase; IC_50_: half maximal inhibitory concentration. *Average of six cell lines.

**Figure 8.**
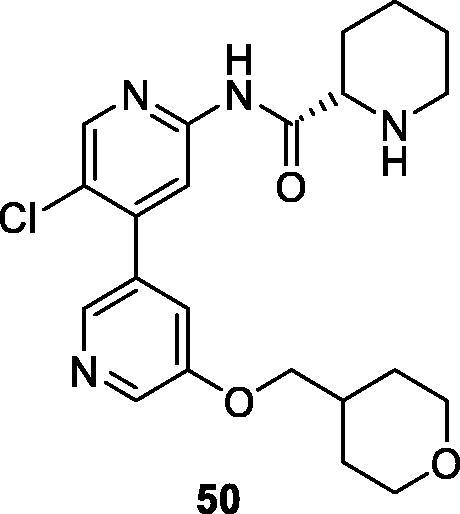
A 2-aminotriazine CDK9 inhibitor published by Novartis.

In 2012, Novartis filed a patent presents 17 compounds with 4-pyridin-3yl-pyridine bearing a 2-carboxyamide group (example **50**; Figure 8). Most of these compounds show CDK9 IC_50_ values below 8 nM^77^.

### Pyrrol[2,3-b]pyridines

**Figure 9.**
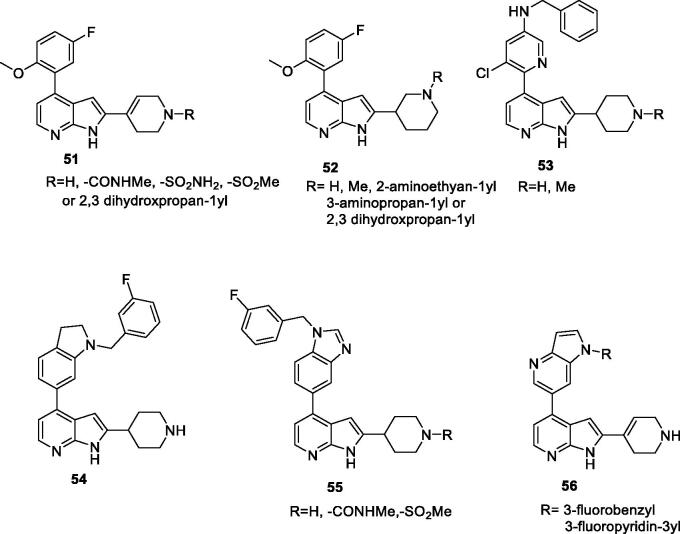
Pyrrolo[2,3-b]pyridines published by AbbVie.

AbbVie published seven patent applications from 2014 to 2016 covering more than 13,600 derivatives of the pyrrolo[2,3-b]pyridine scaffold as CDK9 inhibitors targeting cancer. Examples of the most interesting compounds are illustrated in (general structures **51**–**53**, Figure 9 and Table 9). They inhibit CDK9 with IC_50_ values from 4 to 37 nM. In cell viability assay against A431 and H929 cell lines, they exhibit IC_50_ values between 17 and 280 nM. In cell western blots (used to measure phosphorylation of RNA polymerase II), IC_50_ values are between 26 and 170 nM. In H929 human multiple myeloma cancer xenograft model in mice, they exhibit up to 83% tumour growth inhibition with a high dose (30 mg/kg) and up to 63% with a low dose (1.8 mg/kg) administered intraperitoneally^78^^,^^79^. In additional patents, Lai et al. disclosed 56 related pyrrolopyridines with general structures **54**–**56**. All derivatives were assayed in biochemical assay against CDK9; IC_50_ values are between 11 and 180 nM. The most potent derivatives exhibit single-digit nanomolar cytotoxicity EC_50_ in A431 and H929 cell lines. The most interesting examples exhibit antitumour activity in an H929 human multiple myeloma cancer xenograft model, inhibiting tumour growth up to 25% and delaying the tumour growth up to 5% with a 3.75 mg/kg intraperitoneal dose^80^.

**Table 9.** Pyrrolo[2,3-b]pyridines published by AbbVie.

Scaffold	Selected example R*	CDK9 IC_50_ (nM)	Additional information	Ref
**51**	Methylsulfonyl	37	Cell-viability IC_50_ in A431 and H929 are 0.089 and 0.13 µM, respectively. In cell western blots, the IC_50_ to reduce phosphorylation of RNA polymerase II is 0.16 µM. In a H929 xenograft model in mice, the compound exhibits 66% total growth inhibition with a 15 mg/kg dose.	^78^ ^,^ ^79^
**52**	H	16	Cell-viability IC_50_ in A431 and H929 are 0.89 and 0.28 µM, respectively. In cell western blots, the IC_50_ to reduce phosphorylation of RNA polymerase II is 0.085 µM. In a H929 xenograft model in mice, the compound shows 72% total growth inhibition with a 12.5 mg/kg dose.	^78^ ^,^ ^79^
**53**	H	21	Cell-viability IC_50_ in A431 and H929 are 0.025 and 0.077 µM, respectively. In cell western blots, the IC_50_ to reduce phosphorylation of RNA polymerase II is 0.17 µM. In a H929 xenograft model in mice, the compound shows 60% total growth inhibition with a 5 mg/kg dose.	^79^
**54**	–	64	Cell-viability IC_50_ in A431 and H929 are 0.55 and 0.49 µM, respectively. In a H929 xenograft model in mice, the compound shows 66% total growth inhibition with a 3.75 mg/kg dose.	^80^
**55**	H	4	Cell-viability EC_50_ in H929 is 0.8 µM. In a H929 xenograft model in mice, the compound shows 39% total growth inhibition with 7.5 mg/kg dose.	^80^
**56**	3-fluroropyridn-3yl	14	Cell-viability EC_50_ in A431 and H929 are 0.91 and 0.09 µM, respectively.	^80^

CDK: cyclin-dependent kinase; EC_50_: half maximal effective concentration; IC_50_: half maximal inhibitory concentration. *From the corresponding general structures in Figure 9.

**Figure 10.**
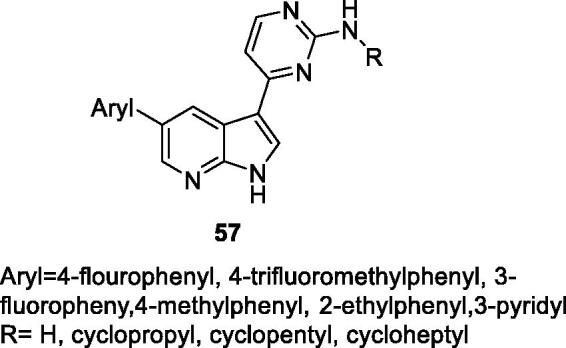
Pyrrolo[2,3-b]pyridines published by the Council of Scientific and Industrial Research.

In 2017, Singh et al. at the Council of Scientific and Industrial Research claimed pyrrolopyridines for the treatment of disorders associated with inappropriate CDK9 activity. A total of 100 compounds were synthesised and assessed against CDK9 and CDK2 (general structure **57**; Figure 10). No specific biological values were disclosed, but the most potent inhibitors claimed to have >70% inhibition at 500 nM in *in vitro* CDK9 assay and >70% inhibition at 10 µM in a proliferation assay against five cell lines, including the prostate cancer cell lines PC-3^81^.

**Figure 11.**
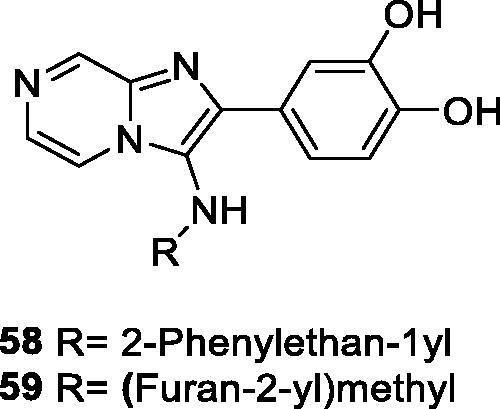
Imidazo[1,2-a]pyrazines published by the University of Ulsan.

### Other bicyclic compounds

Ki et al. at the University of Ulsan claimed the natural products Imidazo[1,2-a]pyrazines **58** and **59** (patent in Korean) as CDK9 inhibitors, with a specific use in breast cancer. The target hits where identified by virtual search. The *in vitro* CDK9 IC_50_ values of **58** and **59** are 7.88 and 5.12 µM, respectively. In proliferative assay in six human breast cancer cell lines, **58** and **59** show cytotoxicity EC_50_ from 29 to 89 µM^82^ (Figure 11).

AbbVie filed a patent on pyrrolo[2,3-d]pyrimidines as CDK9 inhibitors. The patent describes the synthesis of 87 derivatives, their activity in *in vitro* kinase assay and viability data in A431 and H929 cell lines. Representative examples (**60**–**63**; Table 10) show two-digit nanomolar IC_50_ values against CDK9 and an average EC_50_ of 0.11 µM in a cytotoxicity assay against A431 and H929 cell lines^83^.

**Table 10.** Pyrrolo[2,3-d]pyrimidines published by AbbVie.

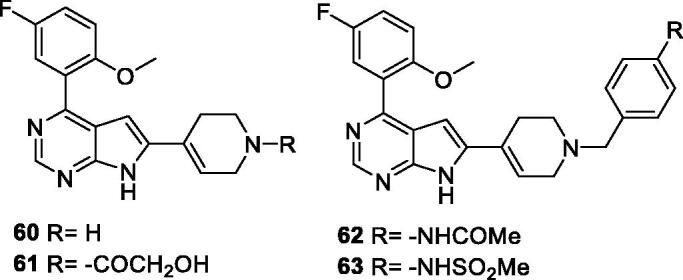		
Compound	CDK9 IC_50_ (nM)	Cytotoxicity* EC_50_ (µM)
**60**	13	0.116
**61**	37	0.165
**62**	76	0.058
**63**	75	0.105

CDK: cyclin-dependent kinase; EC_50_: half maximal effective concentration; IC_50_: half maximal inhibitory concentration. *Average of 2 cell lines.

**Figure 12.**
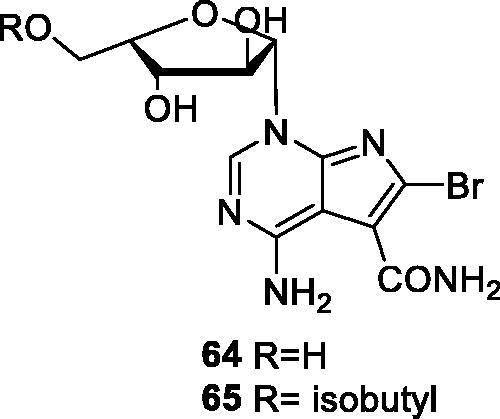
Pyrrolo[2,3-d]pyrimidine-5-carboxamide published by the SUN R&D Foundation.

In 2013, the SNU R&D Foundation disclosed pyrrolo[2,3-d]pyrimidine-5-carboxamide derivatives for the prevention or treatment of liver cancer. The most interesting derivatives, compounds **64** and **65** (shown in Figure 12), inhibit CDK9 with 1 and 40% residual activity, respectively, at 100 nM. They also show some inhibitory activity against other CDK isoforms, including CDK1, CDK2 and CDK7. In western blotting experiments, both compounds effectively inhibit the phosphorylation of RNA polymerase II Ser2 with 1.6% residual activity at 10 nM. Compound **65** successfully downregulates Mcl-1, survivin and XIAP in SNU-354 cells (hepatocellular carcinoma cell line). Moreover, this reduction is attributed to mRNA, confirmed by real-time polymerase chain reaction (RT-PCR). Cell viability was measured by MTT assay in SNU-354 cells. The results showed that the cell viability with **64** and **65** is 36.6 and 16.8% residual activity at 50 µM, respectively. This inhibitory effect on cell growth is greater than that of the standard CDK inhibitors olomoucine and roscovitine. Compounds **64** and **65** induce apoptosis of SNU-354 cells, confirmed by a PARP cleavage experiment. In particular, **65** induces PARP cleavage after 6 h. Furthermore, both compounds increase caspase activity in a dose-dependent manner. In animal studies using a SNU-354 cancer xenograft model, **65** reduces the tumour growth by 15% at 4 mg/kg/day and 47% at 20 mg/kg/day^84^.

In 2015, Bondke et al. at Cancer Research Technology filed a patent on pyrazolo[1,5-a]pyrimidine-5,7-diamine scaffold as CDK inhibitors for treatment of proliferative disorders. Compound **66** inhibits CDK9 with an IC_50_ of 1.1 µM, but it also strongly inhibits CDK7 other CDK isoforms, including CDK2 and CDK1 (Table 11). In a cell growth inhibition assay, compound **66** inhibits the growth of breast cancer cell line (MCF7) and colorectal cancer cell line (HTC116) with an IC_50_ <1 µM. In an HCT116 tumour xenograft model, it reduces the tumour growth by 65% with 100 mg/kg daily dose^85^. In a separate publication in collaboration with Carrick Therapeutics, the same group disclosed **67** mainly as a CDK7 inhibitor, but it also strongly inhibits CDK9 (Table 11). No further biological data were provided in the above mentioned publication^86^.

**Table 11.** Pyrazolo[1,5-a]pyrimidines published by Cancer Research Technology.

			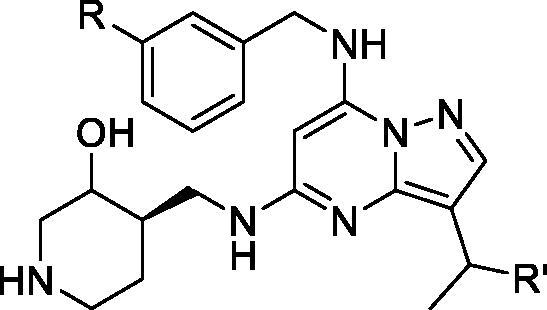				
Compound	R	R′	CDK9 IC_50_ (µM)	CDK7 IC_50_ (µM)	CDK2 IC_50_ (µM)	CDK1 IC_50_ (µM)	Cytotoxicity* IC_50_ (µM)
**66**	H	Me	1.1	0.041	0.58	1.52	0.98
**67**	–CN	H	0.022	0.0089	0.19	ND	ND

CDK: cyclin-dependent kinase; IC_50_: half maximal inhibitory concentration; ND: not determined. *Average of two cell lines.

**Figure 13.**
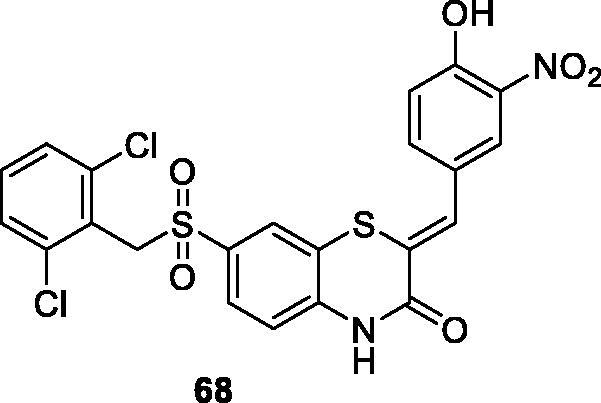
Benzothiazines published by Temple University.

Temple University filed a patent application on CDK9 inhibitors focussed on a benzothiazin-3-one core. The authors claimed compound **68** (Figure 13) as a CDK9 inhibitor for treatment of cancer, with a CDK9 IC_50_ value of 93.7 nM. In an anti-proliferative assay, it shows selective toxicity to cancer cell lines over normal human stem cells, with an average IC_50_ of 0.26 µM in eight cancer cell lines, including prostate cancer, pancreatic cancer, breast cancer and leukaemia^87^.

### Chromones

In 2019, researchers at China Pharmaceutical University filed a patent on a series of flavonoids as antitumour agents (patent in Chinese). The most potent derivatives, **69** and **70**, are claimed to inhibit the growth of HepG2, A549, HCT116 and THP-1 cancer cell lines, with IC_50_ values from 0.4 to 4.4 µM. The researchers claimed that both compounds are monospecific CDK9 inhibitors with low nanomolar potency and selectivity greater than 50 fold over other CDK isoforms (Table 12). In addition, **69** and **70** show reasonable *in vitro* physiochemical properties, including aqueous solubility and lipophilicity. In MV4-11 cancer cells, **69** induces apoptosis in a concentration-dependent manner; it exhibits 40% apoptosis at 1 µM. In a western blot assay in MV4-11 cell lines, **69** inhibits CDK9 activity by reducing the expression of RNA polymerase II and induces apoptosis in an Mcl-1 dependent manner. It also shows sustained induction of cleaved caspase-3 in MV4-11 cells^88^.

**Table 12.** Flavonoids published by China Pharmaceutical University.

	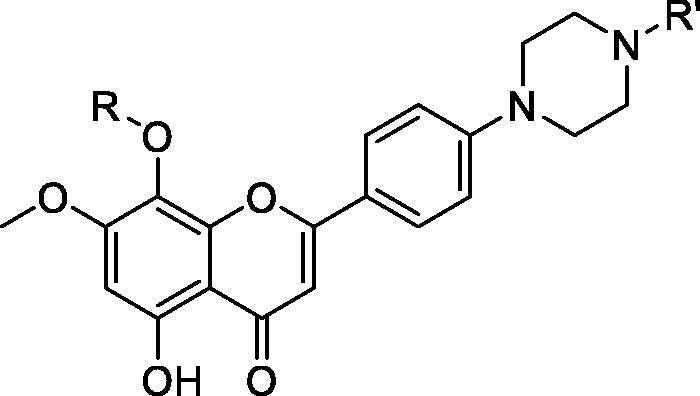	
Compound	**69**	**70**
R	3,5-Dimethyl-1H-pyrazol-4-yl	Piperazin-1yl
R′	Me	H
IC_50_ (nM)		
CDK9	2	3
CDK1	432	ND
CDK2	910	1180
CDK4	254	ND
CDK7	56	NAD
Cytotoxicity*	1400	1800

CDK: cyclin-dependent kinase; IC_50_: half maximal inhibitory concentration; ND: not determined. *Average of 11 cell lines.

The Council of Scientific and Industrial Research filed a patent on chromones related to rohitukine [5,7-dihyroxy-8-[3-hydroxy-1-methylpiperidin-4-yl]-4H-chromen-4-ones] analogues for treating or preventing proliferative disorders. The most potent inhibitors (**71**–**73**) inhibit CDK9 with IC_50_ values between 2 and 30 nM. They also show a CDK2 inhibitory effect, with IC_50_ values between 16 and 608 nM. In cytotoxicity assay in a panel of cell lines, including HL-60, PC3, A-375, MIAPaCa-2, MCF-7 and Caco-2, they show anti-proliferative activity at micromolar concentrations. In *in vivo* animal studies, a Ehrlich solid tumour mouse model, compound **71** inhibits 37% tumour growth with a 70 mg/kg/day intraperitoneal dose without mortality. Compounds **71** and **72** show good aqueous solubility (>1500 µg/mL)^89^ (Table 13).

**Table 13.** Chromones published by the Council of Scientific and Industrial Research.

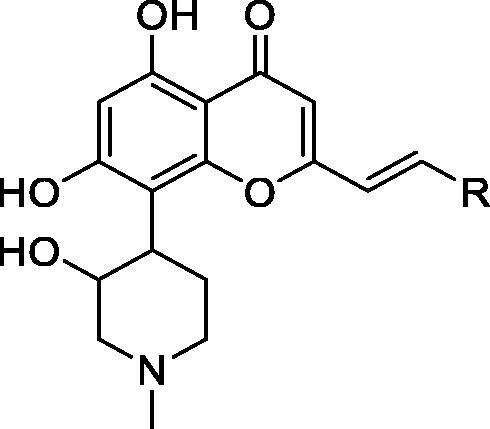			
Compound	**71**	**72**	**73**
*R*	2,6-Dichlorophenyl	2,3,4,5,6-Tetrafluorophenyl	3-Fluorophenyl
CDK9/T1 IC_50_ (nM)	2	12	30
CDK2/A IC_50_ (nM)	16	608	466
Cytotoxicity * IC_50_ (µM)	12.2	10.4	ND

CDK: cyclin-dependent kinase; IC_50_: half maximal inhibitory concentration; ND: not determined. *Average of five cell lines.

### Thiazoles

**Figure 14.**
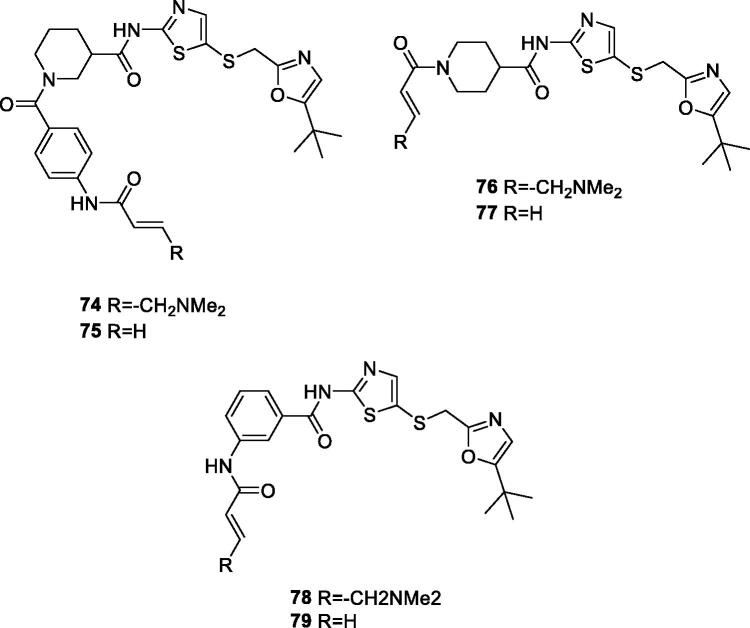
Thiazoles published by the Dana–Farber Cancer Institute.

Nathanael et al. at the Dana–Farber Cancer Institute disclosed several 2-((thiazol-5-ylthio)methyl)oxazole as CDK inhibitors for treatment of cancer. Selected examples are shown in Table 14. They exhibit very low nanomolar IC_50_ against CDK9. They also inhibit other isoforms, including CDK2, CDK7, CDK12 and CDK13 which could contribute to their anti-proliferative activity^90^ (Figure 14).

**Table 14.** Thiazoles published by the Dana–Farber Cancer Institute.

Compound*	74	75	76	77	78	79
IC_50_ (nM)						
CDK9	0.495	3.16	4.4	1.79	10.7	24.6
CDK2	11.5	10.8	23.2	11	29	58.8
CDK7	132	457	82.5	99	226	1140

CDK: cyclin-dependent kinase; IC_50_: half maximal inhibitory concentration. *From the corresponding structures in Figure 14.

**Figure 15.**
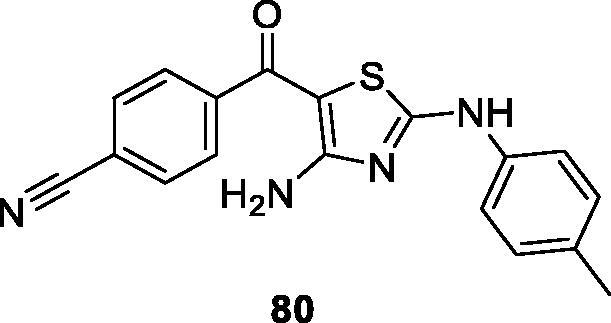
A diaminothiazole compound published by Apogee Biotechnology.

Smith et al. at Apogee Biotechnology filed a patent for diaminothiazole derivatives as anti-proliferative and anti-inflammatory agents. Compound **80** shows a CDK9 IC_50_ value of 0.32 µM. It also has some activity against GSK3β, SK1 and SK2. It shows sub-micromolar IC_50_ against prostatic and pancreatic cell lines. Compound **80** arrests the cancer cells in the G2/M phase of the cell cycle in Pan02 and Hep3B cells when treated with 3 µM of the inhibitor for 18 h. It also demonstrates a loss of the microtubule network. In *in vivo* animal studies using a murine pancreatic cancer Pan02 xenograft model, compound **80** reduces the tumour growth by 70% at dose of 5 mg/kg/day^91^ (Figure 15).

### Macrocyclic compounds

**Figure 16.**
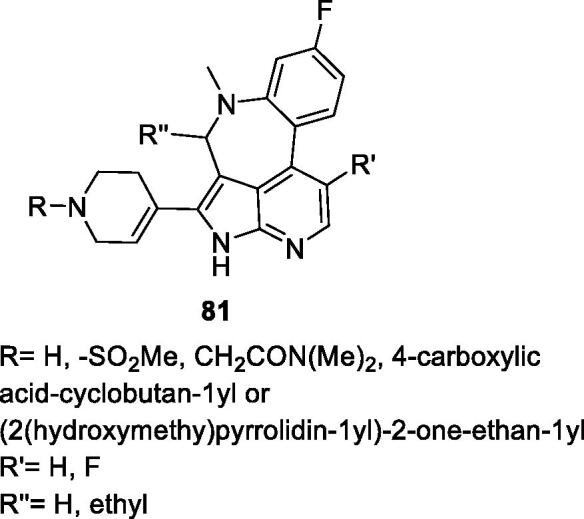
Tetracyclic compounds published by AbbVie.

In 2015, Frey et al. at AbbVie explored several tetracyclic systems as CDK9 inhibitors for cancer treatment. A total of 193 compounds were designed and prepared with general scaffold **81** presented in Figure 16. Many of these compounds inhibit CDK9 with an IC_50_ between 0.021 and 1 µM and cytotoxicity IC_50_ between 0.006 and 5 µM in H929 cells (Table 15)^92^.

**Table 15.** Tetracyclic compounds published by AbbVie.

Selected examples*			CDK9 IC_50_ (nM)	Cytotoxicity H929 IC_50_ (µM)
*R*	*R*′	*Rʺ*		
SO_2_Me	H	ethyl	120	0.032
CH_2_CON(Me)_2_	H	ethyl	60	0.008
(2(hydroxymethy)pyrrolidin-1yl) -2-one-ethan-1yl	F	H	51	0.008

CDK, cyclin-dependent kinase; IC_50_, half maximal inhibitory concentration. *From the corresponding general structure in Figure 16.

In 2019, G1 Therapeutics disclosed pyrrolo[2,3-d]pyrimidines with a spirocyclic fragment as CDK inhibitors. The most interesting compounds (**82**–**84**, Table 16) inhibit CDK9 with low nanomolar IC_50_ values and selectivity greater than 200 fold over nine other CDK isoforms. The authors did not provide additional biological data in the above-mentioned publications^93–95^.

**Table 16.** Macrocyclic pyrrolo[2,3-d]pyrimidines published by G1 Therapeutics.

	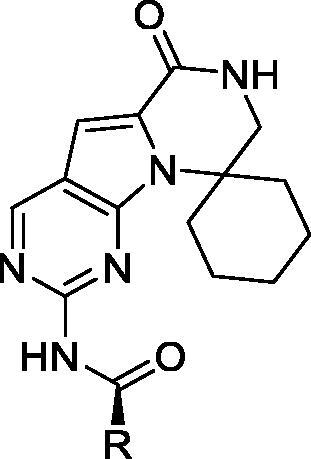		
Compound	82	83	84
R	Piperidin-3yl	Pyrrolodin-3yl	Morpholin-2yl
IC_50_ (µM)			
CDK9/T	0.0055	0.012	0.088
CDK7/H	13	12	61
CDK6/D3	28	14	>100
CDK5/p25	35	19	43
CDK5/p35	30	16	40
CDK1/B1	4.4	5.6	31
CDK2/A	3.1	3.1	10
CDK2/E	13	12	26
CDK3/E	7.2	8.6	54
CDK4/D1	2.9	2.3	27

CDK: cyclin-dependent kinase; IC_50_: half maximal inhibitory concentration.

## Summary and perspective

CDK9 plays a central role in transcription through phosphorylation of RNA polymerase II. At the present time, there is strong evidence that implicates CDK9 as a cancer target. Its anticancer mechanism is believed to be through promotion of short-lived anti-apoptotic genes such as Mcl-1 and Myc. Targeting CDK9 has received great academic and industrial interest. AbbVie, Bayer and Novartis have been the main contributors to the CDK9 patent literature. 2-Aminopyridines/pyrimidines and pyrrolo[2,3-b]pyridines/pyrimidines are the main chemical motifs for CDK9 inhibitors. (Supplementary Table 1 is a list of the CDK9 patents published from 2012 to date classified by chemical type and applicant). Numerous potent and selective CDK9 inhibitors have been disclosed with single-digit nanomolar potency and selectivity greater than 30 fold over other CDK isoforms (e.g. 2-aminopyridines **9** and **10**, 2-aminopyrimidines **33** and **40**, 2-aminotriazine **47** and flavonoids **69** and **70**).

Clinical applications of early pan-CDK9 inhibitors (**1**–**5**) have yielded unambiguous positive results that have mainly been attributed to the lack the selectivity. Therefore, pharmaceutical companies have focussed on developing mono-specific CDK9 inhibitors over the last decade. Despite the challenge in targeting a single CDK isoform due to the high structural similarity of the active sites among homologous kinase isoforms, it has been possible to selectively inhibit CDK9 using a number of chemical scaffolds. Indeed, three selective CDK9 clinical agents (**6**–**8**) are currently being evaluated in human trials, but no results have been disclosed yet.

On the other hand, targeting multiple survival pathways by pan or dual inhibitors can be interesting clinically. A number of *in vitro* studies support the above statement, such as the reported synergism between CDK9 and BRD4 inhibition to reduce Myc expression and dual inhibition of CDK9-mediated Mcl-1 and PI3K-mediated Bcl-xL^96^^,^^97^. Moreover, there have been positive clinical outcomes with some pan-kinase inhibitors, e.g. palbociclib, initially developed as a selective CDK4/6 inhibitor. However, a recent study has shown that it is also a potent CDK9 inhibitor and engages with several lipid kinases^98^. Such evidence will argue against the prevailing view of removing all off-target effects. Additional preclinical and clinical studies are required to determine whether selectively targeting CDK9 will lead to better cancer therapy.

## Supplementary Material

Supplemental Material

## References

[1] Malumbres M, Barbacid M. Cell cycle, CDKs and cancer: a changing paradigm. Nat Rev Cancer 2009;9:153–66. 19238148 10.1038/nrc2602

[2] Yik JHN, Chen R, Nishimura R, et al. Inhibition of P-TEFb (CDK9/cyclin T) kinase and AQRNA polymerase II transcription by the coordinated actions of HEXIM1 and 7SK snRNA. Mol. Cell 2003;12:971–82. 14580347 10.1016/s1097-2765(03)00388-5

[3] Baumli S, Lolli G, Lowe ED, et al. The structure of P-TEFb (CDK9/cyclin T1), its complex with flavopiridol and regulation by phosphorylation. Embo J 2008;27:1907–18. 18566585 10.1038/emboj.2008.121 PMC2486423

[4] Morales F, Giordano A. Overview of CDK9 as a target in cancer research. Cell Cycle 2016;15:519–27. 26766294 10.1080/15384101.2016.1138186 PMC5056610

[5] Huang CH, Lujambio A, Zuber J, et al. CDK9-mediated transcription elongation is required for MYC addiction in hepatocellular carcinoma. Genes Dev 2014;28:1800–14. 25128497 10.1101/gad.244368.114 PMC4197965

[6] Gordon V, Bhadel S, Wunderlich W, et al. CDK9 regulates AR promoter selectivity and cell growth through serine 81 phosphorylation. Mol Endocrinol 2010;24:2267–80. 20980437 10.1210/me.2010-0238 PMC2999477

[7] Rahaman MH, Kumarasiri M, Mekonnen LB, et al. Targeting CDK9 : a promising therapeutic opportunity in prostate cancer. Endocr Relat Cancer 2016;23:T211–226. 27582311 10.1530/ERC-16-0299

[8] Kretz AL, Schaum M, Richter J, et al. CDK9 is a prognostic marker and therapeutic target in pancreatic cancer. Tumor Biol 2017;39:101042831769430. 10.1177/1010428317694304 28231737

[9] Franco LC, Morales F, Boffo S, Giordano A. CDK9: A key player in cancer and other diseases. J Cell Biochem 2018;119:1273–84. 28722178 10.1002/jcb.26293

[10] Wang J, Dean DC, Hornicek FJ, et al. Cyclin-dependent kinase 9 (CDK9) is a novel prognostic marker and therapeutic target in ovarian cancer. Faseb J 2019;33:5990–6000. 30726104 10.1096/fj.201801789RR PMC6463912

[11] Boffo S, Damato A, Alfano L, Giordano A. CDK9 inhibitors in acute myeloid leukemia. J Exp Clin Cancer Res 2018;37:36. 29471852 10.1186/s13046-018-0704-8 PMC5824552

[12] Krystof V, Baumli S, Furst R. Perspective of cyclin-dependent kinase 9 (CDK9) as a drug target. Curr Pharm Des 2012;18:2883–90. 22571657 10.2174/138161212800672750 PMC3382371

[13] Glaser SP, Lee EF, Trounson E, et al. Anti-apoptotic mcl-1 is essential for the development and sustained growth of acute myeloid leukemia. Genes Dev 2012;26:120–5. 22279045 10.1101/gad.182980.111 PMC3273836

[14] Mueller D, García-Cuéllar MP, Bach C, et al. Misguided transcriptional elongation causes mixed lineage leukemia. PLOS Biol 2009;7:e1000249. 19956800 10.1371/journal.pbio.1000249 PMC2774266

[15] Pawar A, Gollavilli PN, Wang S, Asangani IA. Resistance to BET inhibitor leads to alternative therapeutic vulnerabilities in castration-resistant prostate cancer. Cell Rep 2018;22:2236–45. 29490263 10.1016/j.celrep.2018.02.011

[16] Johnstone CN, Mongroo PS, Rich AS, et al. Parvin-beta inhibits breast cancer tumorigenicity and promotes CDK9-mediated peroxisome proliferator-activated receptor gamma 1 phosphorylation. Mol Cell Biol 2008;28:687–704. 17998334 10.1128/MCB.01617-06 PMC2223422

[17] Wang L, Gao W, Hu F, et al. MicroRNA-874 inhibits cell proliferation and induces apoptosis in human breast cancer by targeting CDK9. FEBS Lett 2014;588:4527–35. 25281924 10.1016/j.febslet.2014.09.035

[18] Mitra P, Yang RM, Sutton J, et al. CDK9 inhibitors selectively target estrogen receptor-positive breast cancer cells through combined inhibition of MYB and MCL-1 expression. Oncotarget 2016;7:9069–83. 26812885 10.18632/oncotarget.6997 PMC4891027

[19] Senderowicz AM. Flavopiridol: the first cyclin-dependent kinase inhibitor in human clinical trials. Invest New Drugs 1999;3:313–20. 10.1023/a:1006353008903 10665481

[20] Kumar SK, LaPlant B, Chng WJ, Mayo Phase 2 Consortium, et al. Dinaciclib, a novel CDK inhibitor, demonstrates encouraging single-agent activity in patients with relapsed multiple myeloma. Blood 2015;125:443–8. 25395429 10.1182/blood-2014-05-573741 PMC4296007

[21] Tong W, Chen R, Plunkett W, et al. Phase I and pharmacologic study of SNS-032, a potent and selective Cdk2, 7, and 9 inhibitor, in patients with advanced chronic lymphocytic leukemia and multiple myeloma. J Clin Oncol 2010;28:3015–22. 20479412 10.1200/JCO.2009.26.1347 PMC4979218

[22] Van Der Biessen DAJ, Burger H, De Bruijn P, et al. Phase I study of RGB-286638, a novel, multitargeted cyclin-dependent kinase inhibitor in patients with solid tumors. Clin Cancer Res 2014;20:4776–84. 25024258 10.1158/1078-0432.CCR-14-0325

[23] Walsby E, Pratt G, Shao H, et al. A novel Cdk9 inhibitor preferentially targets tumor cells and synergizes with fludarabine. Oncotarget 2014;5:375–85. 24495868 10.18632/oncotarget.1568 PMC3964214

[24] Zhai S, Senderowicz AM, Sausville EA, Figg WD. Flavopiridol, a novel cyclin-dependent kinase inhibitor, in clinical development. Ann Pharmacother 2002;36:905–11. 11978170 10.1345/aph.1A162

[25] McInnes C. Progress in the evaluation of CDK inhibitors as anti-tumor agents. Drug Discov Today 2008;13:875–81. 18639646 10.1016/j.drudis.2008.06.012

[26] Lücking U, Scholz A, Lienau P, et al. Identification of atuveciclib (BAY 1143572), the first highly selective, clinical PTEFb/CDK9 inhibitor for the treatment of cancer. ChemMedChem 2017;12:1776–93. 28961375 10.1002/cmdc.201700447 PMC5698704

[27] Cidado J, Boiko S, Proia T, et al. AZD4573 is a highly selective CDK9 inhibitor that suppresses Mcl-1 and induces apoptosis in hematologic cancer cells. Clin Cancer Res 2020;26:922–34. 31699827 10.1158/1078-0432.CCR-19-1853

[28] Sonawane YA, Taylor MA, Napoleon JV, et al. Cyclin dependent kinase 9 inhibitors for cancer therapy. J Med Chem 2016;59:8667–8684. 27171036 10.1021/acs.jmedchem.6b00150 PMC5636177

[29] Byth KF, Thomas A, Hughes G, et al. AZD5438, a potent oral inhibitor of cyclin-dependent kinases 1, 2, and 9, leads to pharmacodynamic changes and potent antitumor effects in human tumor xenografts. Mol Cancer Ther 2009;8:1856–1866. 19509270 10.1158/1535-7163.MCT-08-0836

[30] Mariaule G, Belmont P. Cyclin-dependent kinase inhibitors as marketed anticancer drugs: Where are we now? A short survey. Molecules 2014;19:14366–14382. 25215591 10.3390/molecules190914366 PMC6271685

[31] Parry D, Guzi T, Shanahan F, et al. Dinaciclib (SCH 727965), a novel and potent cyclin-dependent kinase inhibitor. Mol Cancer Ther 2010;9:2344–2353. 20663931 10.1158/1535-7163.MCT-10-0324

[32] Conroy A, Stockett DE, Walker D, et al. SNS-032 is a potent and selective CDK 2, 7 and 9 inhibitor that drives target modulation in patient samples. Cancer Chemother Pharmacol 2009;64:723–32. 19169685 10.1007/s00280-008-0921-5

[33] Cirstea D, Hideshima T, Santo L, et al. Small-molecule multi-targeted kinase inhibitor RGB-286638 triggers P53-dependent and -independent anti-multiple myeloma activity through inhibition of transcriptional CDKs. Leukemia 2013;27:2366–2375. 23807770 10.1038/leu.2013.194 PMC3928098

[34] T Parrott, M Weller, TM Estok, E Le Rhun. P08.32 TG02, an oral CDK inhibitor, demonstrates activity in glioma models: EORTC Brain Tumor Group conducts phase 1b study (STEAM/EORTC 1608). Neuro Oncol 2016;18(Suppl 4):iv48.

[35] Goh KC, Novotny-Diermayr V, Hart S, et al. TG02, a novel oral multi-kinase inhibitor of CDKs, JAK2 and FLT3 with potent anti-leukemic properties. Leukemia 2012;26:236–243. 21860433 10.1038/leu.2011.218

[36] Byrne M, Frattini MG, Ottmann OG, et al. Phase I study of the PTEFb inhibitor BAY 1251152 in patients with acute myelogenous leukemia. Blood 2018;132:4055–4055.

[37] Chao SH, Price DH. Flavopiridol inactivates P-TEFb and blocks most RNA polymerase II transcription *in vivo*. J Biol Chem 2001;276:31793–9. 11431468 10.1074/jbc.M102306200

[38] Lin TS, Ruppert AS, Johnson AJ, et al. Phase II study of flavopiridol in relapsed chronic lymphocytic leukemia demonstrating high response rates in genetically high-risk disease. J Clin Oncol 2009;27:6012–8. 19826119 10.1200/JCO.2009.22.6944 PMC2793044

[39] Karp JE, Garrett-Mayer E, Estey EH, et al. Randomized phase II study of two schedules of flavopiridol given as timed sequential therapy with cytosine arabinoside and mitoxantrone for adults with newly diagnosed, poor-risk acute myelogenous leukemia. Haematologica 2012;97:1736–42. 22733022 10.3324/haematol.2012.062539 PMC3487449

[40] Johnson AJ, Yeh YY, Smith LL, et al. The novel cyclin-dependent kinase inhibitor dinaciclib (SCH727965) promotes apoptosis and abrogates microenvironmental cytokine protection in chronic lymphocytic leukemia cells. Leukemia 2012;26:2554–7. 22791353 10.1038/leu.2012.144 PMC3645353

[41] Gojo I, Sadowska M, Walker A, et al. Clinical and laboratory studies of the novel cyclin-dependent kinase inhibitor dinaciclib (SCH 727965) in acute leukemias. Cancer Chemother Pharmacol 2013;72:897–908. 23949430 10.1007/s00280-013-2249-z PMC3784060

[42] Stephenson JJ, Nemunaitis J, Joy AA, et al. Randomized phase 2 study of the cyclin-dependent kinase inhibitor dinaciclib (MK-7965) versus erlotinib in patients with non-small cell lung cancer. Lung Cancer 2014;83:219–23. 24388167 10.1016/j.lungcan.2013.11.020

[43] Mita MM, Joy AA, Mita A, et al. Randomized phase II trial of the cyclin-dependent kinase inhibitor Dinaciclib (MK-7965) versus capecitabine in patients with advanced breast cancer. Clin Breast Cancer 2014;14:169–76. 24393852 10.1016/j.clbc.2013.10.016

[44] Walsby E, Lazenby M, Pepper C, Burnett AK. The cyclin-dependent kinase inhibitor SNS-032 has single agent activity in AML cells and is highly synergistic with cytarabine. Leukemia 2011;25:411–9. 21212792 10.1038/leu.2010.290

[45] Chen R, Wierda WG, Chubb S, et al. Mechanism of action of SNS-032, a novel cyclin-dependent kinase inhibitor, in chronic lymphocytic leukemia. Blood 2009;113:4637–45. 19234140 10.1182/blood-2008-12-190256 PMC2680368

[46] Wu J, Yuan Y, Cordova C, et al. Phase I trial of TG02 plus dose-dense or metronomic temozolomide for recurrent anaplastic astrocytoma and glioblastoma in adults. J Clin Oncol 2019;37:2031.

[47] Narita T, Ishida T, Ito A, et al. Cyclin-dependent kinase 9 is a novel specific molecular target in adult T-cell leukemia/lymphoma. Blood 2017;130:1114–1124. 28646117 10.1182/blood-2016-09-741983

[48] Scholz A, Oellerich T, Hussain A, et al. Abstract 3022: BAY 1143572, a first-in-class, highly selective, potent and orally available inhibitor of PTEFb/CDK9 currently in Phase I, shows convincing anti-tumor activity in preclinical models of acute myeloid leukemia (AML). Cancer Res 2016;76:3022–3022.

[49] Luecking UT, Scholz A, Kosemund D, et al. Abstract 984: Identification of potent and highly selective PTEFb inhibitor BAY 1251152 for the treatment of cancer: from p.o. to i.v. application via scaffold hops. Cancer Res 2017;77:984–984.

[50] Cidado J, Proia T, Boiko S, et al. Abstract 310: AZD4573, a novel CDK9 inhibitor, rapidly induces cell death in hematological tumor models through depletion of Mcl1. Cancer Res 2018;78:310–310.

[51] Rule S, Kater AP, Brümmendorf TH, et al. A phase 1, open-label, multicenter, non-randomized study to assess the safety, tolerability, pharmacokinetics, and preliminary antitumor activity of AZD4573, a potent and selective CDK9 inhibitor, in subjects with relapsed or refractory hematological mali. J Clin Oncol 2018;36:TPS7588–TPS7588.

[52] Wang B, Wu J, Wu Y, et al. Discovery of 4-(((4-(5-chloro-2-(((1s,4s)-4-((2-methoxyethyl)amino)cyclohexyl)amino)pyridin-4-yl)thiazol-2-yl)amino)methyl)tetrahydro-2H-pyran-4-carbonitrile (JSH-150) as a novel highly selective and potent CDK9 kinase inhibitor. Eur J Med Chem 2018;158:896–916. 30253346 10.1016/j.ejmech.2018.09.025

[53] Zhou G, GenFleet Therapeutics Inc. Novel inhibitor of cyclin-dependent kinase CDK9, Patent WO2018192273; 2018.

[54] Rahaman MH, Yu Y, Zhong L, et al. CDKI-73: an orally bioavailable and highly efficacious CDK9 inhibitor against acute myeloid leukemia. Invest. New Drugs 2019;37:625–635. 30194564 10.1007/s10637-018-0661-2

[55] Wang S, Shao H., Changzhou Le Sun Pharmaceuticals Ltd. Therapeutic compounds, Patent WO2013156780; 2013.

[56] Si J, Jiang M, Yang Z, Zhang L., Ancureall Pharmaceutical (Shanghai) Co Ltd., Pyrimidine compound, preparation method thereof and medical thereof, Patent WO2019154177; 2019.

[57] Lücking U, Hog D, Christ C, et al., Bayer. Novel PTEFb inhibiting macrocyclic compounds, Patent WO2018177899; 2018.

[58] Lücking U, Geisler J, Hog D, et al., Bayer. Novel macrocyclic sulfondiimine compounds, Patent WO2017055196; 2017.

[59] Kosemund D, Lücking U, Siemeister G, et al., Bayer. Sulfoximine substituted 5-fluoro-n-(pyridin-2-yl)pyridin-2-amine, Patent WO2015001021; 2015.

[60] Lücking U, Scholz A, Lienau P, et al., Bayer. Disubstituted 5-fluoro pyrimidine derivatives containing a sulfondiimine group, Patent WO2015150273; 2015.

[61] Kosemund D, Lücking U, Scholz A, et al., Bayer. 5-Fluoro-N-(pyridin-2-yl)pyridin-2-amine derivatives containing a sulfone group, Patent WO2015136028; 2015.

[62] Lücking U, Bohlmann R, Kosemund D, et al., Bayer. 4- (Ortho) -fluorophenyl-5-fluoropyrimidin-2-ylamine containing a sulfone group, Patent WO2013037896; 2014.

[63] Lücking U, Kosemund D, Scholz A, et al., Bayer. Disubstituted 5-fluoro-pyrimidines, Patent WO2013037896; 2013.

[64] Lücking U, Böhnke N, Scholz A, et al. 5-Fluoro-N-(pyridin-2-yl)pyridin-2-amine derivatives containing a sulfoximine group, Patent WO2014076091; 2014.

[65] Kosemund D, Lücking U, Siemeister G, et al. Bayer. Fluorinated benzofuranyl-pyrimidine derivatives containing a sulfone group, Patent WO2016059011; 2016.

[66] Kosemund D, Lücking U, Siemeister G, et al., Bayer. Fluorinated benzofuranyl-pyrimidine derivatives containing a sulfoximine group, Patent WO2016059086; 2016.

[67] Wang S, Al Haj Diab S, Long Y., Aucentra Therapeutics Pty Ltd. Derivatives of n-cycloalkyl/heterocycloalkyl-4-(imidazo [1,2-a]pyridine)pyrimidin-2-amine as therapeutic agents, Patent WO2018141002; 2018.

[68] Antonios-Mccrea WR, Barsanti PA, Hu C, et al., Novartis. Phenyl-heteroaryl amine compounds and their uses, Patent WO2012066065; 2012.

[69] Antonios-Mccrea WR, Barsanti PA, Hu C, et al., Novartis. Substituted bi-heteroaryl compounds as CDK9 inhibitors and their uses, Patent WO2012101062; 2012.

[70] Antonios-Mccrea WR, Barsanti PA, Hu C, et al., Novartis. Pyrimidine biaryl amine compounds and their uses, Patent WO2012101065; 2012.

[71] R Antonios-Mccrea WR, Barsanti PA, Hu C, et al., Novartis. 3-(Aminoaryl)-pyridine compounds, Patent WO2012066070; 2012.

[72] Lori F, Chafouleas J, De Forni D, et al., Novel. 4,6-Disubstituted aminopyrimidine derivatives, Patent WO2014031937; 2014.

[73] Lai C, Mastracchio A, Miyashiro JM, et al., AbbVie Inc. Pyridine CDK9 kinase inhibitors, Patent WO2014160017; 2014.

[74] Pike G, Kurt , Barlaam C, et al., AstraZeneca AB. Polycyclic amide derivatives used as CDK9 inhibitors, Patent WO2017001354; 2017.

[75] Choidas A, Klebl B, Habenberger P, et al., Lead Discovery Center. CDK9 inhibitors in the treatment of midline carcinoma, Patent WO2013026874; 2013.

[76] Lücking U, Bohlmann R, Scholz A, et al., Bayer. 4-Aryl-N-phenyl-1,3,5-triazin-2-amines containing a sulfoximine group, Patent WO2012160034; 2012.

[77] Barsanti PA, Hu C, Jin X, et al., Novartis. N-acyl pyridine biaryl compounds and their uses, Patent WO2012101063; 2012.

[78] Tong Y, Bruncko M, Clark RF, et al., AbbVie Inc. Pyrrolo [2,3-B] pyridine CDK9 kinase inhibitors, Patent WO2014139328; 2014.

[79] Gong JU, Tao Z-FU, Tong YU, et al., AbbVie Inc. Pyrrolo[2,3-b]pyridine cdk9 kinase inhibitors, Patent WO2014151444; 2014.

[80] Lai C, Mastracchio A, Miyashiro JM, et al., AbbVie Inc. Pyridine CDK9 kinase inhibitors, Patent WO2014159999; 2014.

[81] Singh U, Mahajan G, Chashoo G, et al., Council of Scientific and Industrial Research. 3-Pyrimidinyl pyrrolo [2,3-b] pyridine as new anticancer agents and the process for the preparation thereof, Patent WO2017094026; 2017.

[82] Kim IK, Kikim I, Nam KY, et al., University of Ulsan Foundation for Industry Cooperation. Composition for prevention and treatment of cancer including CDK9 inhibitor as active ingredient, Patent KR1020180106188; 2019.

[83] Florjancic AS, Tong Y, Penning TD, et al., AbbVie Inc. Substituted pyrrolo[2,3-d]pyrimindines as CDK9 kinase inhibitors, Patent WO2014160028; 2014.

[84] Lee SK, Kim BM, Cho SJ, Kim YJ., SNU R&DB Foundation. CDK-inhibiting pyrrolopyrimidinone carboxamide derivative or a pharmaceutically acceptable salt thereof, and a pharmaceutical composition containing same as an active ingredient for preventing or treating liver cell cancer, Patent WO2012060482; 2012.

[85] Bondke A, Kroll S, Barrett A, et al., Cancer Research Technology Ltd. Pyrazolo[1,5-a]pyrimidine-5,7-diamine compounds as CDK inhibitors and their therapeutic use, Patent WO2015124941; 2015.

[86] Bahl A, Ainscow E, Bondke A, et al., Carrick Therapeutics Ltd, Cancer Research Technology Ltd. Imperial Innovations Ltd.,4-[[(7-aminopyrazolo[1,5-a]pyrimidin-5-yl)amino]methyl]piperidin-3-ol compounds as CDK inhibitors, Patent WO2019057825; 2019.

[87] P. Reddy E, R. Reddy MV., Temple University-of the Commonwealth System of Higher Education. Substituted 2-benzylidene-2h-benzo[b] [1,4]τηιazin-3(4h)-ones, derivatives thereof, and therapeutic uses thereof, Patent WO2012166586; 2012.

[88] Jinlei B, Zhiyu L, Tengteng Z, et al., China Pharmaceutical University. Novel CDK9 inhibitor based on benzofuran structure, preparation method and application thereof, Patent CN110028475; 2019.

[89] Asrey VR, Bibishan BS, Shashi B, et al., Council for Scientific and Industrial Research. Rohitukine analogs as cyclin-dependent kinase inhibitors and a process for the preparation, Patent WO2014170914; 2014.

[90] Gray S, Nathanael T, Zhang P, et al., Dana-Farber Cancer Institute. Inhibitors of cyclin-dependent kinases, Patent WO2017044858; 2017.

[91] Smith D, Charles Y, Zhuang W, Maines L, Apogee Biotechnology Corporation. Diaminothiazole compounds, composition and methods of use, Patent WO2018089902; 2018.

[92] Frey R, Gong J, Ji Z, et al., AbbVie Inc. Tetracyclic CDK9 kinase inhibitors, Patent WO2015119712; 2015.

[93] Strum JC, Jung D, G1 Therapeutics Inc. Heterocyclic compounds for the treatment of abnormal cellular proliferation, Patent WO2019136244. 2019.

[94] Strum JC, G1 Therapeutics Inc. CDK inhibitors for the treatment of neoplastic, Patent WO2019222521; 2019.

[95] Strum JC, G1 Therapeutics Inc. Pyrimidine-based compounds for the treatment of cancer, Patent WO2018005863; 2018.

[96] Thomas D, Powell JA, Vergez F, et al. Targeting acute myeloid leukemia by dual inhibition of PI3K signaling and Cdk9-mediated Mcl-1 transcription. Blood 2013;122:738–748. 23775716 10.1182/blood-2012-08-447441

[97] Lu H, Xue Y, Yu GK, et al. Compensatory induction of MYC expression by sustained CDK9 inhibition via a BRD4-dependent mechanism. Elife 2015;4:1–26. 10.7554/eLife.06535 PMC4490784 26083714

[98] Sumi NJ, Kuenzi BM, Knezevic CE, et al. Chemoproteomics reveals novel protein and lipid kinase targets of clinical CDK4/6 inhibitors in lung cancer. ACS Chem Biol 2015;10:2680–2686. 26390342 10.1021/acschembio.5b00368 PMC4684772

