# Supplementary material for: Small molecule inhibitors of cyclin-dependent kinase 9 for cancer therapy
Source: J Enzyme Inhib Med Chem. 2021 Feb 25;36(1):693–706. doi: 10.1080/14756366.2021.1890726 (PMC7919902; doi:10.1080/14756366.2021.1890726)
Supplement: Supplemental Material [file IENZ_A_1890726_SM0802.pdf]

Supplementary Table s1: List of CDK9 patents in proliferative disorders

Chemical scafold	Applicant	Patent title	Patent number
2-Aminopyridines	GenFleet Therapeutics	Novel inhibitor of cyclin-dependent kinase CDK9	WO2018192273
2-Aminopyrimidines	Changzhou Le Sun Pharmaceuticals	Therapeutic compounds	WO2013156780
2-Aminopyrimidines	Ancureall Pharmaceutical	Pyrimidine compound, preparation method thereof and medical thereof	WO2019154177
2-Aminopyrimidines	Bayer	4- (Ortho) -fluorophenyl-5-fluoropyrimidin-2-ylamine containing a sulfone group	WO2014060376
2-Aminopyrimidines	Bayer	Disubstituted 5-fluoro pyrimidine derivatives containing a sulfondiimine group	WO2015150273
2-Aminopyrimidines	Bayer	Disubstituted 5-Fluoro-pyrimidines	WO2013037896
2-Aminopyridines	Bayer	Novel PTEFb inhibiting macrocyclic compounds	WO2018177899
2-Aminopyridines/pyrimidines	Bayer	Novel macrocyclic sulfondiiminc compounds	WO2017055196
2-Aminopyridines	Bayer	5-Fluoro-N-(pyridin-2-yl)pyridin-2-amine derivatives containing a sulfone group	WO2015136028
2-Aminopyridines	Bayer	5-Fluoro-N-(pyridin-2-yl)pyridin-2-amine derivatives containing a sulfoximine group	WO2014076091
2-Aminopyrimidines	Bayer	Fluorinated benzofuranyl-pyrimidine derivatives containing a sulfone group	WO2016059011
2-Aminopyrimidines	Bayer	Fluorinated benzofuranyl-pyrimidine derivatives containing a sulfoximine group	WO2016059086
2-Aminopyridines	Bayer	Sulfoximine substituted 5-fluoro-n-(pyridin-2-yl)pyridin-2-amine	WO2015001021
2-Aminopyrimidines	Aucentra Therapeutics	Derivatives of n-cycloalkyl/heterocycloalkyl-4-(imidazo [1,2-a]pyridine)pyrimidin-2-amine as therapeutic agents	WO2018141002
4-phenyl-2-aminopyridines	Novartis	Phenyl-heteroaryl amine compounds and their uses	WO2012066065
Biaryle compounds	Novartis	Substituted bi-heteroaryl compounds as CDK9 inhibitors and their Uses	WO2012101062
4-pyridin-4-yl-2-aminopyrimidines	Novartis	Pyrimidine biaryl amine compounds and their uses	WO2012101065
2-aminopyridines	Novartis	3-(Aminoaryl)-Pyridine Compounds	WO2012066070
4-Aminopyrimidines	ViroStatics	Novel4,6-disubstituted aminopyrimidine derivatives	WO2014031937
Pyridines amide	AbbVie	Pyridine CDK9 kinase inhibitors	WO2014160017
Pyridines/pyrimidines amide	AstraZeneca	Polycyclic amide derivatives used as CDK9 inhibitors	WO2017001354
2-Aminotriazines	Lead Discovery Center	CDK9 inhibitors in the treatment of midline carcinoma	WO2013026874
2-Aminotriazines	Bayer	4-Aryl-N-phenyl-1,3,5-triazin-2-amines containing a sulfoximine group,	WO2012160034
4-Pyridin-3yl-pyridines	Novartis	N-acyl pyridine biaryl compounds and their uses	WO2012101063
Pyrrolo [2,3-b]pyridines	AbbVie	Pyrrolo [2,3-B] pyridine CDK9 kinase inhibitors	WO2014139328
Pyrrolo [2,3-b]pyridines	AbbVie	Pyrrolo[2,3-b]pyridine cdk9 kinase inhibitors	WO2014151444
Pyrrolo [2,3-b] pyridines	AbbVie	Pyridine CDK9 kinase inhibitors	WO2014159999
Pyrrolo [2,3-b] pyridines	Council of Scientific and Industrial Research	3-Pyrimidinyl pyrrolo [2,3-b] pyridine as new anticancer agents and the process for the preparation thereof	WO2017094026
Imidazo[1,2-a]pyrazines	University of Ulsan Foundation for	Composition for Composition for prevention and treatment of cancer including	KR1020180106188

	Industry Cooperation	CDK9 inhibitor as active ingredient	
pyrrolo[2,3-d]pyrimindines	AbbVie	Substituted pyrrolo[2,3-d]pyrimindines as CDK9 kinase inhibitors,	WO2014160028
pyrrolo[2,3-d]pyrimidines	SNU R&DB Foundation	CDK-inhibiting pyrrolopyrimidinone carboxamide derivative or a pharmaceutically acceptable salt thereof, and a pharmaceutical composition containing same as an active ingredient for preventing or treating liver cell cancer	WO2012060482
Pyrazolo[1,5-a]pyrimidines	Cancer Research Technology	Pyrazolo[1,5-a]pyrimidine-5,7-diamine compounds as CDK inhibitors and their therapeutic use, WO2015124941	WO2015124941
Pyrazolo[1,5-a]pyrimidines	Carrick Therapeutics, Cancer Research Technology	4-[[(7-Aminopyrazolo[1,5-a]pyrimidin-5-yl)amino]methyl]piperidin-3-ol compounds as CDK inhibitors	WO2019057825
Benzothiazines	Temple University of the commonwealth system of higher education	Substituted 2-benzylidene-2h-benzo[b] [1,4]τηιazin-3(4h)-ones, derivatives thereof, and therapeutic uses thereof	WO2012166586
Flavonoids	China Pharmaceutical University	Novel CDK9 inhibitor based on benzofuran structure, preparation method and application thereof	CN110028475
Chromones	Council for Scientific and Industrial Research	Rohitukine analogs as cyclin-dependent kinase inhibitors and a process for the preparation	WO2014170914
Thiazoles	Dana-Farber Cancer Institute	Inhibitors of cyclin-dependent kinases	WO2017044858
Diaminothiazoles	Apogee	Diaminothiazole compounds, composition and methods of use	WO2018089902
Tetracyclic compounds	AbbVie	Tetracyclic CDK9 Kinase Inhibitors	WO2015119712
Macrocyclic pyrrolo[2,3-d]pyrimidines	G1 Therapeutics	Heterocyclic compounds for the treatment of abnormal cellular proliferation	WO2019136244
Macrocyclic pyrrolo[2,3-d]pyrimidines	G1 Therapeutics	CDK inhibitors for the treatment of neoplastic	WO2019222521
Macrocyclic pyrrolo[2,3-d]pyrimidines	G1 Therapeutics	Pyrimidine-based compounds for the treatment of cancer	WO2018005863
